# External validation of a bifactor model of oppositional defiant disorder

**DOI:** 10.1038/s41380-018-0294-z

**Published:** 2018-12-11

**Authors:** Irwin D. Waldman, Richard Rowe, Khrista Boylan, Jeffrey D. Burke

**Affiliations:** 1 Department of Psychology, Emory University, 475 PAIS Building, 36 Eagle Row, Atlanta, GA 30322 USA; 2 Department of Psychology, University of Sheffield, Sheffield, UK; 3 Department of Psychology, McMaster University, Hamilton, ON Canada; 4 Department of Psychology, University of Connecticut, Storrs, CT USA

**Keywords:** Psychology, Genetics

## Abstract

Dimensions of irritability and defiant behavior, though correlated within the structure of ODD, convey separable developmental risks through adolescence and adulthood. Irritability predicts depression and anxiety, whereas defiant behavior is a precursor to antisocial outcomes. Previously we demonstrated that a bifactor model comprising irritability and defiant behavior dimensions, in addition to a general factor, provided the best-fitting structure of ODD symptoms in five large datasets. Herein we extend our previous work by externally validating the bifactor model of ODD using multiple regression and multivariate behavior genetic analyses. We used parent ratings of DSM IV ODD symptoms, and symptom dimensions for ADHD (i.e., inattention and hyperactivity−impulsivity), conduct disorder (CD), depression/dysthymia, and generalized anxiety disorder (GAD) from 846 6−18-year-old twin pairs. We found that the ODD irritability factor was associated only with depression/dysthymia and GAD and the ODD defiant behavior factor was associated only with inattention, hyperactivity−impulsivity, and CD, whereas the ODD general factor was associated with all five symptom dimensions. Multivariate behavior genetic analyses found all five symptom dimensions shared genetic influences in common with the ODD general, irritability, and defiant behavior factors. In contrast, the defiant behavior factor shared genetic influences uniquely with inattention and hyperactivity−impulsivity, whereas the irritability factor shared genetic influences uniquely with depression/dysthymia and GAD, but not vice versa. This suggests that genes that influence irritability in early childhood also predispose to depression and anxiety in adolescence and adulthood. These multivariate genetic findings also support the external validity of the three ODD dimensions at the etiological level. Our study provides additional support for subtyping ODD based on these symptom dimensions, as in the revisions in the ICD-11, and suggests potential mechanisms underlying the development from ODD to behavioral or affective disorders.

## Introduction

The presence of oppositional defiant disorder (ODD) in young people confers the risk for a wide range of future psychopathology in later adolescence and adulthood [1]. While links to later conduct disorder (CD) are well established, ODD also predicts anxiety and depression. These associations are particularly remarkable as they have been shown to exist independently from comorbid CD, with CD conferring no additional risk independently from ODD. Studies of ODD symptoms have provided evidence for distinct dimensions of irritability versus defiant behavior [1–6], as reflected in revisions to DSM 5 [7] and the International Classification of Diseases (ICD) [8]. Irritability robustly predicts later depression and anxiety [1, 5, 6, 9, 10], but not later attention deficit hyperactivity disorder (ADHD) [11], CD [12], substance use [6], bipolar disorder symptoms [11], or borderline personality disorder [13, 14]. Furthermore, irritability can be validly measured early in childhood [15, 16], and intergenerational links have been observed between preschool irritability and parental history of depression, suicidality and anxiety [15, 16], but not parental antisocial behavior or substance use [15, 16]. The convergent and discriminant validity of irritability as a meaningfully distinct dimension from defiant behavior is thus supported both over individuals’ developmental lifespans and inter-generationally.

Several studies demonstrate moderate genetic influences on irritability, with heritability estimates ranging from 31 to 54% across studies in the UK [17], Sweden [18, 19], and USA [20]. Moderate heritability estimates for defiant behavior, ranging from 41 to 45%, also have been reported in these studies, with moderate shared environmental influences as well. In the UK sample, the genetic correlation of irritability was stronger with depressed mood (*r*_A_ = .70) than with delinquency (*r*_A_ = .57), whereas the genetic correlation of defiant behavior was stronger with delinquency (*r*_A_ = .80) than with depressed mood (*r*_A_ = .46). In the US sample, irritability at age 11 shared genetic influences with internalizing symptomatology at age 16, whereas defiant behavior at age 11 shared genetic influences with later substance use disorder symptoms. The longitudinal Swedish study [18, 19] found that the covariation of irritability with internalizing symptoms accounted for by genetic influences ranged from 56 to 74% across waves. In order to more fully describe the specificity of the genetic contribution to the phenotypic associations between irritability and internalizing disorders and between defiant behavior and externalizing behavioral disorders, it is necessary to account for *common* genetic factors shared between the specific ODD dimensions and other psychiatric disorders. Evidence for a genetic pathway linking childhood irritability with later depression or anxiety would have profound implications for the early identification of affective disorder risk, given the potential to identify irritability during preschool.

### The present study

Our previous work demonstrated that a bifactor structure including both a general ODD factor and specific irritability and defiant behavior factors provided the best fit to ODD symptoms in five large datasets [1], including the Georgia Twin Study (GTS), the sample used herein. In the GTS we operationalized the general factor using all symptoms, irritability using the temper, touchy, and angry symptoms, and defiant behavior using the argues, defies, annoys, blames, and spiteful symptoms. In this paper we first explore the external validity of the general and specific (irritability and defiant) ODD factors by testing their differential phenotypic associations with contemporaneous symptoms of internalizing psychopathology (depression/dysthymia, generalized anxiety disorder (GAD)) and externalizing psychopathology (CD, inattentive and hyperactive−impulsive ADHD symptom dimensions). We hypothesize that the ODD general factor reflects predispositions to, and thus will have phenotypic associations with, both internalizing and externalizing psychopathology. In contrast, we hypothesize that the specific defiant behavior factor will be more strongly associated with externalizing disorders whereas the specific irritability factor will be more strongly associated with internalizing disorders.

We next capitalize on the genetically informative design of the GTS to estimate the genetic and environmental influences on the ODD factors and their overlap with the internalizing and externalizing symptom dimensions. Previous studies have shown both common and unique genetic influences on externalizing and internalizing symptom dimensions [21]. We hypothesize that there will be substantial common genetic influences on the general ODD factor and all the other forms of psychopathology. Further, we hypothesize that the genetic influences specific to irritability will be shared primarily with internalizing psychopathology, whereas the genetic influences specific to the defiant behavior factor will be shared primarily with externalizing psychopathology. If supported, this will mean that the ODD general factor is important to isolate as it will allow the specific phenotypic and etiological associations of irritability and defiant behaviors with other outcomes to be more clearly studied.

## Materials and methods

### Participants and measures

#### Georgia Twin Study (GTS)

The GTS comprises 846 twin pairs from the Georgia Twin Registry, a population-based registry of twins (*Mean age* = 10.60 years, *SD* *=* 3.20 years, age range = 6−18 years), with 49% males, 82% European Americans, 11% African Americans, 1% Hispanic Americans, and 6% mixed/other ethnicity. The sample includes 392 (46%) monozygotic (MZ) and 454 (54%) dizygotic (DZ) twin pairs. In 1992−1993, using state birth records, 5620 parents of twins born between 1980 and 1991 in Georgia were contacted via mail. Of these, 1567 twin families joined the registry, among which 846 families provided complete ODD symptom ratings.

Symptom ratings were obtained from a parent (typically mothers) using the Emory Diagnostic Rating Scale (EDRS) [22]. The EDRS assesses symptoms of the major DSM–IV childhood psychiatric disorders. Parents rated symptoms of ADHD, ODD, CD, GAD, and depression/dysthymia on a 0–4 scale. Symptom scales based on these items demonstrated high internal consistency in the current sample (*α* = .95, .89, .91, .82, .90, .87, respectively). The EDRS yields ADHD and ODD diagnostic rates similar to population prevalences [22].

Parents of participating children provided written informed consent, and children provided assent, after receiving a complete description of the study. The study was approved by the Emory University IRB.

## Results

### Multiple regression analyses

Models were estimated using Mplus version 7 [23] using the robust maximum likelihood estimator (MLR) given non-normal symptom dimension distributions. Goodness of fit was evaluated using multiple indices, including the chi-square value, the Akaike Information Criterion (AIC), the Bayesian Information Criterion (BIC), the Tucker−Lewis index (TLI), root mean square error of approximation (RMSEA), and the root mean square residual (RMSR) [24]. The acceptability of model fit was based on collectively comparing these fit indices against published guidelines: TLI ≥ 0.95 for excellent fit [25] and between 0.90 and 0.95 for acceptable fit [26]; RMSEA ≤ 0.08 for adequate fit and ≤0.05 for close fit [27]; RMSR ≤ 1.00 for good model fit [28]. The minimum value of the AIC and BIC was used to indicate the best-fitting alternative model [24].

We first examined the relations of the three ODD factors with the CD, inattention, hyperactivity−impulsivity, depression/dysthymia, and GAD symptom dimensions. Table 1 shows the standardized regression coefficients (*β*’s) of each symptom dimension on all three ODD factors and the sex, age, age^2^, sex × age, and sex × age^2^ covariates simultaneously. We also estimated the percentage of variance (i.e., *R*^2^) explained by all three ODD factors considered together. To test for sex differences, we contrasted the fit of a model in which the standardized regression coefficients for the three ODD factors were equated for boys and girls versus a model in which these coefficients varied by sex.

**Table 1:** Summary of external validity analyses of ODD factor scores from best-fitting bifactor model

Dependent variable			Covariates	*β*General	*β*Irr	*β*Def	*R*^2^	*χ*^2^	df	*p*	BIC_=SEX_	BIC_bySEX_	RMSEA (95% CI)	TLI	RMSR
Inattention															
Sex, Age, Age^2^, Sex × Age				**.24**	.08	**.27**	.23	7.3	3	.062	12,015	12,027	.041 (.000−.079)	.963	.016
		95% CIs		[.20−.30]	[−.09 to .25]	[.10−.44]									
Hyperactivity−impulsivity															
Sex, Age				**.24**	−.06	**.48**	.29	9.4	3	.024	11,367	11,375	.049 (.016−.087)	.964	.026
		95% CIs		[.19−.28]	[-.23 to .11]	[.32−.65]									
Conduct disorder															
Sex, Age				**.28**	−.07	**.53**	.36	5.2	3	.157	9730	9736	.029 (.000−.070)	.978	.024
		95% CIs		[.23−.33]	[-.30 to .15]	[.31−.76]									
Depression/dysthymia															
Age				**.18**	**.35**	−.04	.17	1.7	3	.648	10,535	10,555	.000 (.000−.045)	1.011	.010
		95% CIs		[.12−.23]	[.18−.52]	[-.20−.13]									
Generalized anxiety disorder															
Sex, Age, Age^2^				**.20**	**.40**	−.13	.14	11.0	3	.012	11,961	11,967	.055 (.023−.092)	.898	.022
		95% CIs		[.14-.26]	[.22-.57]	[-.30-.04]									
Irritability not															
Equated by sex					.33/.45		.14/.22	1.2	2	.551	11,954		.000 (.000−.058)	1.015	.002
		95% CIs			[.14−.51]/ [.28−.61]										

The *β*’s are the standardized regression coefficients for the regression of each dependent variable on each of the three ODD factors. *R*^2^ is the % of variance accounted for in each dependent variable by all three ODD factors. The fit statistics are for the comparison of a model in which the standardized regression coefficients for the three ODD factors are equated for boys and girls versus a model in which the standardized regression coefficients for the three ODD factors vary by sex. For all of the dependent variables except for GAD and hyperactivity−impulsivity, all three regression coefficients could be equated for boys and girls. For GAD, a model in which the regression coefficient for ODDIrr was larger for girls than for boys fits better, as indicated in the second row of results for GAD. For hyperactivity−impulsivity, models in which the regression coefficients for ODDIrr and ODDDef varied by sex fit better than one in which all coefficients were equated but these are not shown due to space and due to the possibility that these models fit better simply due to chance

Regression coefficients in bold significantly differ from zero

As predicted, the ODD general factor was associated with all five symptom dimensions with β’s suggesting that each standard deviation increase in the ODD general factor was associated with a .18−.28 standard deviation increase in the external validity symptom dimensions. In contrast, the defiant behavior factor was uniquely associated with only the CD and inattentive and hyperactive−impulsive ADHD symptom dimensions (*β*’s = .53, .27, and .48, respectively) but not depression/dysthymia or GAD (*β’*s = −.04 and −.13, respectively). The irritability factor was uniquely associated with only the depression/dysthymia and GAD symptom dimensions (*β’*s = .35 and .40, respectively) but not the CD, inattentive, or hyperactive−impulsive symptom dimensions (*β’*s = −.07, .08, and −.06, respectively). The variance explained by the three ODD factors was 36% in CD, 23% in inattention, 29% in hyperactivity−impulsivity, 17% in depression/dysthymia, and 14% in GAD.

For all dependent variables except GAD and hyperactivity−impulsivity all three ODD factor regression coefficients could be equated for boys and girls. For GAD, a model in which the regression coefficient for irritability was larger for girls than for boys fits better, as indicated in the second row of results for GAD in Table 1. For hyperactivity−impulsivity, models in which the regression coefficients for irritability and defiant behavior varied by sex fit better than models in which all coefficients were equated but these are not shown due to space and the possibility that improved fit is due to chance.

### Univariate behavior genetic analyses

We next conducted a set of univariate behavior genetic analyses to estimate genetic and environmental influences on the three ODD factors and the five external validity symptom dimensions as a prelude to our multivariate behavior genetic modeling. As shown in Table 2, the best-fitting model for the ODD general factor was the ACE model with moderate additive genetic and nonshared environmental influences (.41 and .45, respectively) and modest but significant shared environmental influences (.13). Although a model without shared environmental influences (the AE model) had a lower BIC (3619 versus 3623), all other fit indices favored the ACE model and the estimate of shared environmental influences was significant, thus favoring the ACE over the AE model. In contrast, the best-fitting model for the irritability and defiant behavior factors was the AE model, with moderate additive genetic (.64 and .68, respectively) and nonshared environmental influences (.36 and .32, respectively).

**Table 2:** Summary of univariate model fitting analyses of ODD factor scores from best-fitting bifactor model

Factor	Model	*χ*^2^	df	*p*	AIC	BIC	RMSEA	TLI	RMSR	*s*	*h*^2^	*c*^2^/*d*^2^	*e*^2^
GENERAL													
	**ACE**	**2.3**	**6**	**.893**	**3604**	**3623**	**.000 (.000−.028)**	**1.010**	**.027**		**.41**	**.13**	**.45**
	ADE	3.9	6	.687	3607	3626	.000 (.000−.048)	1.006	.034		.56	.00	.44
	AE	4.6	7	.712	3605	3619	.000 (.000–.044)	1.006	.034		.56	—	.44
	CE	18.0	7	.012	3620	3634	.060 (.026−.095)	.974	.052		—	.43	.57
w/sib int.	AE + *s*	2.7	6	.849	3605	3624	.000 (.000−.034)	1.009	.031	.05^NS^	.46	—	.51
	CE + *s*	Not identified											
	ADE + *s*	2.2	5	.818	3607	3631	.000 (.000−.040)	1.009	.031	.05^NS^	.46	.00	.51
	ACE + *s*	2.1	5	.832	3606	3630	.000 (.000−.039)	1.009	.026	−.08^NS^	.36	.31 ^NS^	.38
Irritability													
	ACE	10.5	6	.104	3990	4009	.041 (.000−.082)	.990	.065		.64	.00 ^NS^	.36
	ADE	12.9	6	.045	3990	4009	.051 (.007−.090)	.985	.065		.63	.01 ^NS^	.36
	**AE**	**12.3**	**7**	**.091**	**3988**	**4002**	**.041 (.000−.079)**	**.990**	**.065**		**.64**	**—**	**.36**
	CE	57.3	7	<.001	4036	4050	.128 (.098−.159)*	.909	.093		—	.46	.54
w/sib int.	AE + *s*	12.9	6	.045	3990	4009	.051 (.007−.090)	.985	.065	−.01^NS^	.66	—	.35
	CE + *s*	49.1	6	<.001	4038	4057	.128 (.096−.162)*	.909	.093	.22	—	.05	.80
	ADE + *s*	10.7	5	.057	3991	4015	.051 (.000− .093)	.985	.065	−.01^NS^	.66	.00 ^NS^	.35
	ACE + *s*	11.6	5	.041	3990	4014	.055 (.011− .097)	.983	.060	−.17^NS^	.46	.44 ^NS^	.24
Defiant behavior													
	ACE	10.8	6	.093	4040	4059	.043 (.000−.083)	.991	.061		.63	.05 ^NS^	.32
	ADE	9.2	6	.164	4040	4059	.035 (.000− .077)	.994	.062		.68	.00	.32
	**AE**	**10.7**	**7**	**.152**	**4038**	**4053**	**.035 (.000−.074)**	**.994**	**.062**		**.68**	**—**	**.32**
	CE	60.0	7	<.001	4092	4106	.131 (.102−.163)*	.911	.088		—	.50	.50
w/sib int.	AE + *s*	11.3	6	.081	4040	4059	.045 (.000−.084)	.990	.062	.01^NS^	.67	—	.33
	CE + *s*	51.5	6	<.001	4094	4113	.131 (.099−.165)*	.911	.088	.24	—	.05	.77
	ADE + *s*	9.4	5	.094	4042	4066	.045 (.000−.088)	.990	.062	.01^NS^	.67	.00^NS^	.33
	ACE + *s*	9.1	5	.106	4041	4064	.043 (.000−.087)	.990	.054	−.17^NS^	.41	.58	.20

The best-fitting model(s) is shown in bold

*significant, *NS* nonsignificant, *T* statistical trend, *A* additive genetic influences, *D* nonadditive genetic influences, *C* shared environmental influences, *E* nonshared environmental influences, *s* sibling interaction/rater contrast

As shown in Table 3, the best-fitting model for inattention and hyperactivity−impulsivity was the AE model, with the addition of the sibling influence/rater contrast parameter *s*. For both inattention and hyperactivity−impulsivity there were substantial additive genetic (.76 and .87, respectively) and nonshared environmental influences (.29 and .19, respectively), as well as rater contrasts (−.15 and −.12). Results were similar for CD, as the best-fitting model was the AE model with the addition of the sibling influence/rater contrast parameter *s*, with moderate additive genetic (.88) and nonshared environmental influences (.17) as well as rater contrast (−.09). For depression/dysthymia and GAD the best-fitting model was again the AE model, with moderate additive genetic (.61 and .70, respectively) and nonshared environmental influences (.39 and .30, respectively).

**Table 3:** Summary of univariate model fitting analyses of external validity symptom dimensions

Factor	Model	*χ*^2^	df	*p*	AIC	BIC	RMSEA	TLI	RMSR	*s*	*h*^2^	*c*^2^/*d*^2^	*e*^2^
CD													
	ACE	3.3	6	.768	10,057	10,077	.000 (.000−.042)	1.001	.068		.78	.00	.22
	ADE	1.1	6	.981	10,046	10,065	.000 (.000−.000)	1.001	.049		.26	.53	.21
	AE	3.9	7	.794	10,055	10,070	.000 (.000−.038)	1.001	.068		.78	—	.22
	CE	45.8	7	<.001	10,209	10,224	.112 (.083−.144)*	.991	.122		—	.49	.51
w/sib int.	AE + ***s***	**0.8**	**6**	**.992**	**10,044**	**10,063**	**.000 (.000−.000)**	**1.001**	**.033**	**−.09**^**T**^	**.88**	**—**	**.17**
	CE + *s*	39.3	7	<.001	10,211	10,231	.112 (.080−.147)*	.991	.122	.26	—	.01	.81
	ADE + *s*	2.2	5	.818	3607	3631	.000 (.000−.040)	1.009	.031	.05^NS^	.46	.00	.51
	ACE + *s*	1.1	5	.955	10,046	10,070	.000 (.000−.000)	1.001	.032	−.13^NS^	.80	.13 ^NS^	.15
Inattention													
	ACE	28.4	6	.0001	12,322	12,341	.092 (.060−.127)*	.896	.114		.51	.00 ^NS^	.49
	ADE	16.9	6	.0096	12,322	12,341	.064 (.029−.101)	.949	.100		.00	.57	.43
	AE	33.1	7	<.0001	12,320	12,334	.092 (.062−.125)*	.896	.114		.51	—	.49
	CE	68.1	7	<.0001	12,372	12,386	.141 (.112−.172)*	.756	.140		—	.28	.72
w/sib int.	AE + ***s***	**15.6**	**6**	**.016**	**12,297**	**12,316**	**.060 (.024−.098)**	**.955**	**.075**	**−.15**	**.76**	**—**	**.29**
	CE + *s*	58.4	6	<.001	12,374	12,393	.141 (.109−.175)*	.756	.140	.22	—	.005	.94
	ADE + *s*	13.0	5	.023	12,299	12,323	.060 (.020−.102)	.955	.075	−.15	.76	.00	.29
	ACE + *s*	17.1	5	.004	12,299	12,323	.074 (.038−.114)	.932	.072	−.21^NS^	.65	.19 ^NS^	.24
Hyperactivity−impulsivity													
	ACE	16.0	6	.014	11,658	11,677	.062 (.026−.099)	.971	.086		.71	.00	.29
	ADE	3.9	6	.684	11,637	11,656	.000 (.000−.048)	1.006	.060		.00	.73	.27
	AE	18.7	7	.009	11,656	11,670	.000 (.062−.096)	.971	.086		.71	—	.29
	CE	85.3	7	<.001	11,773	11,787	.159 (.130−.191)*	.806	.130		—	.41	.59
w/sib int.	AE + ***s***	**2.3**	**6**	**.894**	**11,634**	**11,653**	**.000 (.000−.028)**	**1.011**	**.034**	**−.12**	**.87**	**—**	**.19**
	CE + *s*	73.1	6	<.001	11,775	11,794	.159 (.128−.193)*	.806	.130	.21	—	.006	.87
	ADE + *s*	1.9	5	.865	11,636	11,659	.000 (.000−.035)	1.011	.034	−.12	.87	.00	.19
	ACE + *s*	2.3	5	.810	11,635	11,659	.000 (.000−.041)	1.009	.033	−.17^NS^	.78	.14 ^NS^	.17
Depression/dysthymia													
	ACE	7.8	6	.254	10,591	10,610	.026 (.000−.071)	.987	.156		.61	.00	.39
	ADE	8.5	6	.206	10,589	10,608	.031 (.000−.074)	.982	.156		.41	.22	.38
	**AE**	**9.1**	**7**	**.246**	**10,589**	**10,603**	**.026 (.000−.068)**	**.987**	**.156**		**.61**	**—**	**.39**
	CE	18.1	7	.011	10,639	10,653	.060 (.027−.095)	.930	.166		—	.39	.61
w/sib int.	AE + *s*	8.5	6	.205	10,586	10,605	.031 (.000−.074)	.982	.143	−.06^NS^	.72	—	.32
	CE + *s*	Did not converge											
	ADE + *s*	7.1	5	.216	10,588	10,612	.031 (.000−.078)	.982	.143	−.06^NS^	.72	.00	.32
	ACE + *s*	Did not converge											
GAD													
	ACE	8.3	6	.217	11,883	11,902	.029 (.000−.073)	.993	.123		.70	.00	.30
	ADE	9.2	6	.163	11,883	11,902	.035 (.000−.077)	.990	.123		.64	.07 ^NS^	.30
	**AE**	**9.7**	**7**	**.208**	**11,881**	**11,896**	**.029 (.000−.070)**	**.993**	**.124**		**.70**	**—**	**.30**
	CE	32.8	7	<.001	11,950	11,964	.092 (.061−.124)	.932	.133		—	.47	.53
w/sib int.	AE + *s*	9.1	6	.168	11,881	11,901	.034 (.000−.076)	.991	.115	−.04^NS^	.76	—	.27
	CE + *s*	Did not converge											
	ADE + *s*	7.6	5	.181	11,883	11,907	.034 (.000−.080)	.991	.115	−.04^NS^	.76	.00	.27
	ACE + *s*	Did not converge											

The best-fitting model(s) is shown in bold

*CD* conduct disorder, *GAD* generalized anxiety disorder, *significant, *NS* nonsignificant, *T* statistical trend, *A* additive genetic influences, *D* nonadditive genetic influences, *C* shared environmental influences, *E* nonshared environmental influences, *s* sibling interaction/rater contrast

### Multivariate behavior genetic analyses

**Fig. 1:**
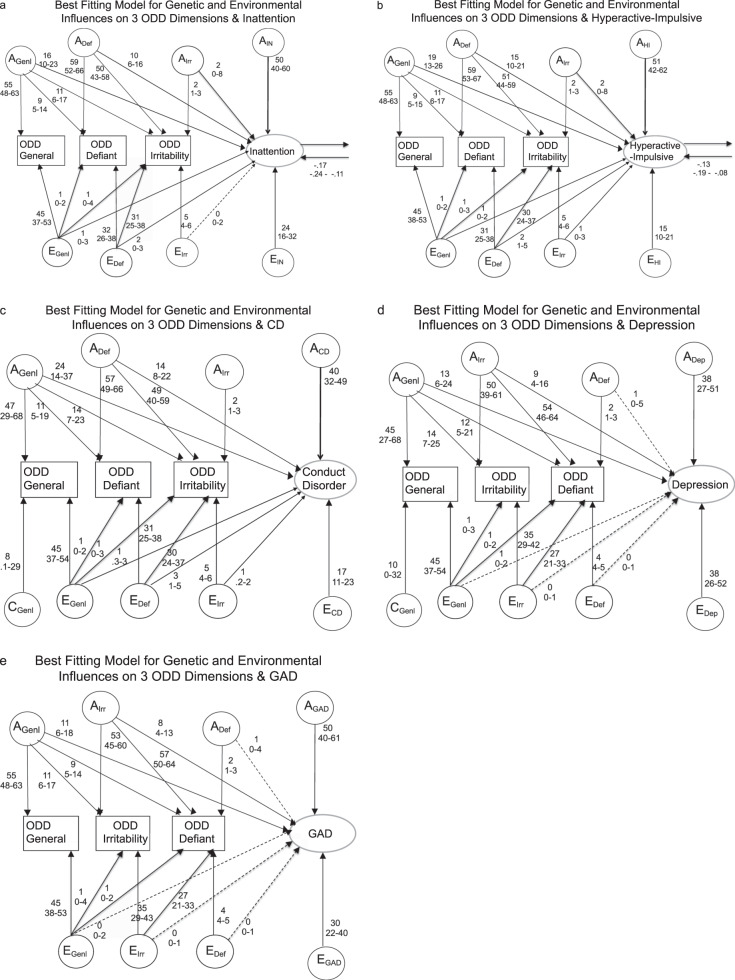
**a** Best fitting model for genetic and environmental influences on three ODD dimensions and inattention. A, additive genetic influences; C, shared environmental influences; E, nonshared environmental influences; IN, inattention; Genl, general factor; Irr, irritability factor; Def, defiant behavior factor. Path coefficients for the genetic and environmental influences on the ODD factors and on inattention are squared standardized regression coefficients (i.e., variance components) with their 95% confidence intervals shown underneath. Decimal points are omitted to save space. **b** Best fitting model for genetic and environmental influences on three ODD dimensions and hyperactivity−impulsivity. A, additive genetic influences; C, shared environmental influences; E, nonshared environmental influences; HI, hyperactivity−impulsivity; Genl, general factor; Irr, irritability factor; Def, defiant behavior factor. Path coefficients for the genetic and environmental influences on the ODD factors and on hyperactivity−impulsivity are squared standardized regression coefficients (i.e., variance components) with their 95% confidence intervals shown underneath. Decimal points are omitted to save space. **c** Best fitting model for genetic and environmental influences on 3 ODD dimensions and CD. A, additive genetic influences; C, shared environmental influences; E, nonshared environmental influences; CD, conduct disorder; Genl, general factor, Irr, irritability factor, Def, defiant behavior factor. Path coefficients for the genetic and environmental influences on the ODD factors and on CD are squared standardized regression coefficients (i.e., variance components) with their 95% confidence intervals shown underneath. Decimal points are omitted to save space. **d** Best fitting model for genetic and environmental influences on three ODD dimensions and depression. A, additive genetic influences; C, shared environmental influences; E, nonshared environmental influences; Dep, depression; Genl, general factor; Irr, irritability factor; Def, defiant behavior factor. Path coefficients for the genetic and environmental influences on the ODD factors and on depression are squared standardized regression coefficients (i.e., variance components) with their 95% confidence intervals shown underneath. Decimal points are omitted to save space. **e** Best fitting model for genetic and environmental influences on three ODD dimensions and GAD. A, additive genetic influences; C, shared environmental influences; E, nonshared environmental influences; GAD, generalized anxiety disorder; Genl, general factor; Irr, irritability factor; Def, defiant behavior factor. Path coefficients for the genetic and environmental influences on the ODD factors and on GAD are squared standardized regression coefficients (i.e., variance components) with their 95% confidence intervals shown underneath. Decimal points are omitted to save space. Dashed lines indicate paths that are not significantly greater than 0

Our multivariate behavior genetic analyses used a series of Cholesky decompositions to model the genetic and environmental influences on the overlap between the three ODD factors and each of the five external validity symptom dimensions (see Table 4 and Fig. 1a–e). In these models the first factor includes all genetic (or environmental) influences that are common to the ODD general and specific factors and the external validity symptom dimension. The second factor includes all genetic (or environmental) influences on defiant behavior that are not shared with the ODD general factor but which also influence the irritability factor and the external validity symptom dimension. The third factor includes genetic (or environmental) influences on irritability that are not shared by the ODD general or defiant behavior factors but which also influence the external validity symptom dimension. The final factor represents genetic (or environmental) influences that are unique to the external validity symptom dimension. Because the ordering of variables is crucial to the interpretation of Cholesky decompositions, we also conducted these analyses switching the order of the defiant behavior and irritability factors. This allows assessment of whether each of these ODD factors shared incremental genetic (or environmental) influences with the external validity symptom dimensions after accounting for the genetic (or environmental) influences that were shared with both of these ODD factors. The effects of shared and nonshared environment were similarly structured.

**Table 4:** Summary of multivariate model fitting analyses of external validity disorders

Factor	Model	*χ*2	df	*p*	AIC	BIC	RMSEA	TLI	RMSR	*A*_Genl_	*A*_Def_	*A*_Irr_	*A*_Res_	*C*_Genl_
Inattention (Order of factors: General, Defiant, Irritability, Inattention)														
1. Full model		Not identified/Did not converge												
1a. Full model (No S on Inattention)		95.9	58	.001	18,864	19,007	.039 (.024−.052)	.992	.054					
2. C only on ODD General		78.6	66	.138	18,816	18,921	.021 (.000−.037)	.998	.051	.18	.09	.02	.49	
3. No C on ODD General		**79.1**	**67**	**.147**	**18,814**	**18,915**	**.020 (.000−.036)**	**.998**	**.052**	**.16**	**.10**	**.02**	**.50**	
4. No General A or E loadings on Def or Irr		256.2	71	<.0001	19,006	19,087	.077 (.067−.087)	.967	.124					
Hyperactivity/impulsivity (Order of factors: General, Defiant, Irritability, Hyperactivity−Impulsivity)														
1. Full model		Not identified/Did not converge												
1a. Full model (No S on Inattention)		75.7	58	.059	18,044	18,187	.026 (.000−.042)	.996	.050					
2. C only on ODD General		57.9	66	.750	17,999	18,104	.000 (.000−.021)	1.001	.040	.22	.13	.02	.40	
3. No C on ODD General		**58.9**	**67**	**.750**	**17,998**	**18,098**	**.000 (.000−.021)**	**1.001**	**.040**	**.19**	**.15**	**.02**	**.51**	
4. No General A or E loadings on Def or Irr		236.6	71	<.0001	18,190	18,271	.073 (.063−.083)	.972	.120					
CD (Order of factors: General, Defiant, Irritability, CD)														
1. Full model		53.9	58	.628	16,276	16,419	.000 (.000−.026)	1.001	.046					
2. C only on ODD General		**62.5**	**67**	**.634**	**16,261**	**16,362**	**.000 (.000−.024)**	**1.001**	**.045**	**.24**	**.14**	**.002**^**NS**^	**.40**	**.08**
3. No C on ODD General		**63.6**	**68**	**.628**	**16,261**	**16,356**	**.000 (.000−.024)**	**1.001**	**.046**	**.20**	**.16**	**.004**^**NS a**^	**.41**	
4. No General A or E loadings on Def or Irr		201.1	72	<.0001	16,455	16,531	.064 (.053−.074)	.979	.120					
Depression/dysthymia (Order of factors: General, Irritability, Defiant, Depression)														
1. Full model		63.8	58	.279	17,264	17,408	.015 (.000–.034)	.999	.070					
2. C only on ODD General		**72.0**	**67**	**.317**	**17,249**	**17,349**	**.013 (.000−.032)**	**.999**	**.070**	**.13**	**.09**	**.01**^**NS**^	**.38**	**.10**
3. No C on ODD General		**73.4**	**68**	**.305**	**17,249**	**17,344**	**.013 (.000−.032)**	**.999**	**.070**	**.11**	**.11**	**.01**^**NS b**^	**.38**	
4. No General A or E loadings on Def or Irr		211.5	72	<.0001	17,443	17,519	.066 (.056−.077)	.975	.128					
GAD (Order of factors: General, Irritability, Defiant, GAD)														
1. Full model		61.0	58	.368	18,615	18,758	.011 (.000−.032)	.999	.057					
2. C only on ODD General		70.6	67	.359	18,602	18,702	.011 (.000−.031)	.999	.059	.12	.07	.005^NS^	.50	.06^NS^
3. No C on ODD General		**71.1**	**68**	**.374**	**18,600**	**18,696**	**.010 (.000−.030)**	**.999**	**.059**	**.11**	**.08**	**.01**^**NS c**^	**.50**	
4. No General A or E loadings on Def or Irr		224.1	72	<.0001	18,793	18,869	.069 (.059−.080)	.972	.125					

Best-fitting models are highlighted in bold

*Def* defiant, *Irr* Irritability, *A*_Res_ residual additive genetic influences on the external validity symptom dimension

^a^Neither Defiant nor Irritability is significant on CD when it is the third factor

^b^Irritability is significant on Depression when it is either the second or third factor

^c^Irritability is significant on GAD when it is either the second or third factor

In the models for CD and depression, the only shared environmental influences were those on the ODD general factor, but these were nonsignificant in the models for inattention, hyperactivity−impulsivity, and GAD. All nonshared environmental influences were significant in the models for inattention, hyperactivity−impulsivity, and CD, but for depression and GAD all of the nonshared environmental influences were unique to those symptom dimensions and none were shared with the ODD factors. The fit statistics for alternative models are shown in Table 4 and the parameter estimates and their 95% confidence intervals from the best-fitting multivariate genetic models are shown in Fig. 1a–e.

**Fig. 2:**
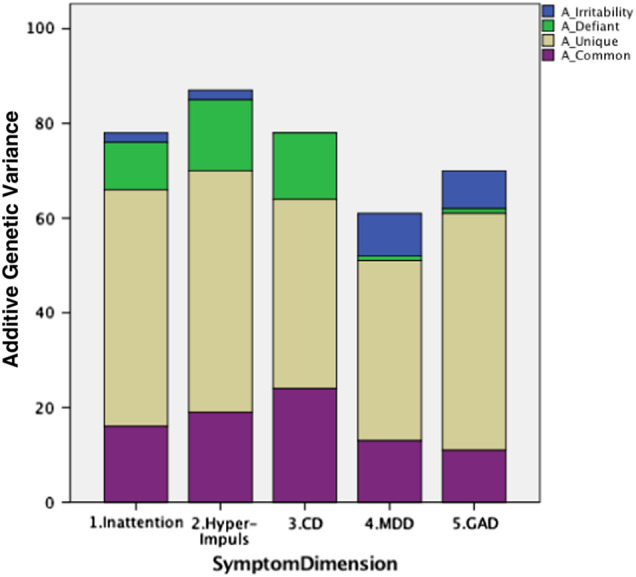
Genetic variance components for external validity symptom dimensions. Hyper-Impuls, hyperactivity−impulsivity; CD, conduct disorder; MDD, major depressive disorder; GAD, generalized anxiety disorder

As shown in Figs. 1a–e and 2, the pattern of additive genetic influences differed across the five symptom dimensions. For inattention and hyperactivity−impulsivity, all genetic influences were significant. This suggests that the genetic influences that these ADHD symptom dimensions share with ODD are common to the general, irritability and defiant behavior factors, with additional residual genetic influences shared with both defiant behavior and irritability, and a final set of genetic influences shared only with irritability. Results were similar for CD, except that there were no genetic influences shared uniquely with irritability. Indeed, neither defiant behavior nor irritability shared unique genetic influences with CD when they were the third factor entered into the analyses.

The majority of the genetic influences that depression/dysthymia and GAD shared with ODD were common to the general, irritability, and defiant behavior factors, although they also shared additional residual genetic influences that were common to both defiant behavior and irritability. In contrast, depression/dysthymia and GAD did not share genetic influences uniquely with defiant behavior, as these were nonsignificant and much lower in magnitude (i.e., <= 1% of the variance). Although each of the five symptom dimensions shared substantial genetic influences in common with the three ODD factors, ranging from 19% for GAD to 38% for CD, the majority of genetic influences were unique to each symptom dimension and were not shared in common with the ODD factors. The breakdown of common and unique genetic influences on each external validity symptom dimension is shown in Fig. 2.

## Discussion

We explored the relations of irritability, defiant behavior, and general ODD factors with other dimensions of externalizing and internalizing psychopathology. This included analyses at the phenotypic and etiological levels, capitalizing on the genetically informative design of the GTS. Both analyses support the external validity of distinguishing ODD symptom dimensions.

Regarding the specific ODD factors, our phenotypic analyses supported our predictions that the irritability factor would uniquely associate with depression and anxiety, but not the externalizing disorders, whereas the disruptive behavior factor would uniquely associate with the other externalizing disorders but not anxiety or depression, replicating previous findings [6, 11, 12, 15, 16, 29–31]. This clear differential pattern of correlates emphasizes the utility of distinguishing these ODD symptom dimensions, even though they typically are highly correlated (e.g. *r*’s = .73 [16], .79 [2], and .91 [1]).

As anticipated, the ODD general factor demonstrated phenotypic associations with all internalizing and externalizing symptom dimensions. It is important to note that our bifactor model specifies orthogonal specific and general factors. Thus, the associations of the internalizing and externalizing symptom dimensions with the ODD general factor were independent of their associations with the specific irritability and defiant behavior factors. This finding suggests that the bifactor modeling approach isolates a meaningful general factor of ODD in the presence of distinct irritability and defiant behavior factors, which cannot be achieved with simpler dimensional models. A key implication of this is that it may be erroneous to separate irritability from defiant behavior as a separate diagnostic category, as has been done with disruptive mood dysregulation disorder in the DSM 5 [7].

Using univariate behavior genetic analyses we tested alternative etiological models of the ODD dimensions. These results using psychiatric symptoms are similar to and extend heritability estimates from previous studies [17, 18]. As in other studies, our modeling indicated that additive genetic rather than shared environmental influences underlie the familial aggregation of irritability and defiant behavior. We estimated moderate heritability (64 and 68%, respectively) for irritability and defiant behavior, estimates that are somewhat higher than those reported elsewhere (e.g., 37 and 45% in Stringaris et al. [17]). This may reflect our modeling of the ODD dimensions using DSM symptoms and as factors within a bifactor framework, with a consequent reduction in measurement error due to using latent factors. Our general ODD factor also showed moderate heritability (41%) and modest shared environmental influences (13%).

We predicted that the differential phenotypic associations between the three ODD factors and the other psychopathology dimensions would be reflected at the etiological level. Multivariate genetic analyses showed substantial overlap between genetic influences on the ODD dimensions and the other dimensions of psychopathology, consistent with findings of common genetic influences on different forms of psychopathology [21, 32]. Consistent with the generalist genes model, we found that the majority of genetic influences that underlie comorbidity were related to the general, irritability, and defiant behavior ODD factors.

The genetic influences on the irritability and defiant behavior factors that were independent from the general ODD factor also contributed to the other forms of psychopathology. We hypothesized that genetic influences specific to the defiant behavior factor would also underlie the externalizing symptom dimensions. As shown in Fig. 2, our results supported this prediction, as CD, inattention, and hyperactivity−impulsivity all shared genetic influences (ranging from 10 to 15% of the variance) in common with defiant behavior, whereas genetic influences shared in common only with irritability were minimal (≤2%) and in the case of CD, nonsignificant. Indeed, CD did not share any genetic influences uniquely with either irritability or defiant behavior, but rather shared genetic influences that were common to both irritability and defiant behavior. This may reflect common genetic influences on all three symptom dimensions shared with negative emotionality [33].

Similarly, as shown in Fig. 2, our prediction that depression and GAD symptoms would share genetic influences uniquely with irritability (ranging from 8 to 9% of the variance) was supported, whereas they did not share genetic influences uniquely with defiant behavior (≤1%). This result is consistent with other behavior genetic analyses, including Stringaris et al.’s [17] finding that the genetic correlation of depression with irritability is higher than with defiant behavior. One explanation for this pattern of results is that genes that underlie the irritability but not the defiant component of ODD increase risk for depressed mood (and generalized anxiety in our study). Thus, the identification of genes underlying the unique association between irritability and depression or anxiety will be obscured when genetic variance shared by irritability, defiant behavior and the general ODD dimension is not distinguished.

A growing body of literature highlights the need to elucidate the hierarchical structure of, and transdiagnostic relations among, different forms of psychopathology. Our results contribute to this emerging literature, and embody these recent trends that emphasize transdiagnostic [34] and hierarchical structural approaches to psychopathology [35–37]. Specifically, our findings validate the distinction between the irritability and defiant behavior ODD symptom dimensions while also furthering the evidence that irritability is correlated with the defiant behavior dimension. Within the heterogeneous ODD construct, shared genetic influences at least partly explain differential phenotypic associations (i.e., irritability with depression and anxiety; defiant behavior with externalizing disorders). The typically early emergence of the ODD phenotype suggests its likely utility for identifying important developmental risk factors for later psychopathology.

### Strengths and limitations

The GTS has a number of advantages for testing the validity and utility of a bifactor model of ODD. These include a large community sample and a genetically informative design. An advantage over extant genetically informative studies of ODD dimensions is our explicit assessment of DSM symptom dimensions. Nonetheless, its cross-sectional design prevents tests of the longitudinal predictive validity of these factors.

Another limitation is the reliance on a single informant for all measures, which leaves correlations vulnerable to inflation due to common method variance [38], such as various rater effects. In bifactor modeling this is most likely to be captured by the general factor, as rater effects would likely be common to all items. In a twin study, where ratings are provided by a single parent, common method variance would be shared by both twins. This is consistent with our findings of shared environmental influences only on the ODD general factor. Given that these effects were small and we did not find shared environmental influences on the other psychopathology dimensions, it seems unlikely that common method variance has influenced our results to an appreciable extent. Nonetheless, future studies using multiple informants will be valuable in further developing the evidence for the external validity of our bifactor modeling approach.
